# Surveillance of travel-associated isolates elucidates the diversity of non-pandemic Vibrio cholerae

**DOI:** 10.1099/mgen.0.001307

**Published:** 2024-10-16

**Authors:** Lia Bote, Alyce Taylor-Brown, Mailis Maes, Danielle J. Ingle, Mary Valcanis, Benjamin P. Howden, Nicholas R. Thomson

**Affiliations:** 1 Wellcome Sanger Institute, Hinxton, UK; 2 The Department of Microbiology and Immunology, The University of Melbourne at The Peter Doherty Institute for Infection and Immunity, Melbourne, Australia; 3 Microbiological Diagnostic Unit Public Health Laboratory, Department of Microbiology & Immunology, The University of Melbourne at The Peter Doherty Institute for Infection and Immunity, Melbourne, Australia; 4 Centre for Pathogen Genomics, The University of Melbourne, Melbourne, Australia; 5 Department of Infectious Diseases and Immunology, Austin Health, Heidelberg, Australia; 6 London School of Hygiene and Tropical Medicine, London, UK

**Keywords:** cholera, surveillance, transmission dynamics, *Vibrio cholerae*, whole-genome sequencing

## Abstract

*Vibrio cholerae* is a Gram-negative bacterium found in aquatic environments and is the aetiological agent of cholera, characterized by acute watery diarrhoea and severe dehydration. Cholera presents a significant global health burden of an estimated 1.3–5 million annual cases, with the current pandemic caused by a toxigenic lineage of the O1 El Tor biotype called seventh pandemic El Tor (7PET) that is still ongoing. Whilst it is known that non-7PET lineages can cause sporadic disease, little is known about the transmission of these non-epidemic lineages. Thirty-four *V. cholerae* isolates were obtained from travellers returning from Indonesia to Australia between 2005 and 2017. These were whole genome sequenced, placed into a global phylogenetic context with 883 isolates, and screened for known genes associated with antimicrobial resistance and virulence. This analysis revealed that 30 isolates fell within non-7PET lineages and four within the 7PET lineage. Both 7PET and non-7PET isolates carried genes for resistance to antibiotics that are commonly used in cholera treatment such as tetracyclines and fluoroquinolones. Diverse virulence factors were also present in non-7PET isolates, with two isolates notably carrying toxin-coregulated pilus genes, which are primarily responsible for intestinal colonization in 7PET *V. cholerae*. This study demonstrates the role of travel in long-range carriage of epidemic and non-epidemic lineages of *V. cholerae,* and how sentinel travel surveillance can enrich our knowledge of *V. cholerae* diversity, reveal new biology about the spread of diverse lineages with differing disease potential and illuminate disease presence in endemic regions with limited surveillance data.

**Impact Statement**

*Vibrio cholerae* remains one of the most important human pathogens globally. While surveillance efforts have focused on the current pandemic lineage, non-epidemic lineages have also been shown to cause sporadic disease. This study demonstrates the ability of diverse epidemic and non-epidemic lineages with virulence and antimicrobial resistance determinants to be carried across long distances, and highlights the importance of sentinel travel surveillance in understanding disease transmission dynamics.

## Data Summary

Sequencing reads were deposited in the NCBI Sequence Read Archive under Bioproject PRJNA856407. Individual accession IDs for each isolate are listed in the Supplementary Data. All other supporting data have been provided within the article or in the supplementary data files.

## Introduction

*Vibrio cholerae* is a Gram-negative, curved or comma-shaped bacterium that is found in marine, coastal or brackish water. It is the causative agent of cholera, which is transmitted through the oral–faecal route and characterized by acute watery diarrhoea and severe dehydration. Cholera represents a significant global health burden of an estimated 1.3–5 million cases and 21 000–143 000 deaths annually [1]. Of the nearly 200 *V*. *cholerae* ‘O’ serogroups based on the O-antigen polysaccharide, only toxigenic strains of O1 and O139 have caused epidemics and global pandemics. Toxigenic *V. cholerae* strains carry the *ctxA* and *ctxB* genes encoding cholera toxin (CTX), which itself is encoded in the genome of the lysogenic CTXφ bacteriophage [2]. CTX is the primary virulence factor linked to secretory diarrhoea. The cell surface receptor for CTXφ is the toxin coregulated pilus (TCP), which is also a key virulence factor essential for intestinal colonization [3].

There have been seven recorded cholera pandemics in history since 1817. The classical biotype of the O1 serogroup was thought to be primarily responsible for the first six and was replaced by a toxigenic, highly clonal lineage of the O1 serogroup, El Tor biotype for the seventh, ongoing pandemic (7PET, seventh pandemic El Tor) [4]. The O139 serogroup is an El Tor derivative that rapidly emerged in 1992 but is no longer linked to outbreaks [5,6]. The 7PET lineage was traced to a source in the Bay of Bengal and has since spread globally through three independent but overlapping waves of transmission [7]. The seventh pandemic itself was first documented in Indonesia in 1961 and continues globally to the present day [7,8]. Despite large cholera outbreaks in the 1970s and 1980s [9,10], and an estimated 0.5 per 1000 annual cases of both O1 and non-O1 *V. cholerae* between 2001 and 2003 [11], the World Health Organization has not reported cholera cases in Indonesia since 2011 [12,13]. Nevertheless, diarrhoea remains a leading cause of mortality for children in Indonesia [1,10].

As per WHO recommendations, while the primary mode of treatment for cholera is oral rehydration therapy, tetracycline is commonly used as the first-line antibiotic due to both efficacy and ease of administration, and fluoroquinolones such as ciprofloxacin are used as alternative treatment [14]. Multidrug resistance (MDR) in 7PET *V. cholerae* is often linked to the acquisition of various mobile genetic elements (MGEs), including the sulfamethoxazole–trimethoprim (SXT) family integrative conjugative element (ICE), which carries genes such as *dfrA1* and *sul1* conferring resistance to trimethoprim and sulfamethoxazole, respectively [15]. This SXT ICE was acquired in 7PET between 1978 and 1984, during the transition between wave 1 and wave 2 of 7PET transmission [7]. The SXT ICE has also been found in non-7PET lineages and can lose the MDR gene cassette as previously seen in O139 strains [4]. As such, the role of other MGEs, such as plasmids and other ICEs, in antimicrobial resistance in *V. cholerae* has also been described [15].

Isolates that do not belong to the 7PET lineage, which are collectively referred to as non-7PET *V. cholerae*, have also been associated with sporadic cases of diarrhoea, gastroenteritis and bacteraemia. These isolates often belong to non-O1, non-O139 serogroups and present with a wider range of symptoms than 7PET infection [16,18]. However, like 7PET, some non-7PET isolates also belong to the O1 serogroup and, although rarely found, CTXφ and TCP are present in some strains [6,19]. In fact, recent cholera outbreaks in China have been attributed to CTX-negative, TCP-positive (CNTP), non-7PET lineages where CTXφ phage acquisition has been observed [20].

Type III secretion systems (T3SSs) have been linked to virulence in other pathogenic Gram-negative bacteria such as *Yersinia*, *Salmonella* and *Shigella*, allowing for the translocation of effectors into eukaryotic host cells [21,22]. These effectors often result in cytoskeletal rearrangements and thus cell perturbation or death. In *Vibrio*, the T3SS was first detected in *V. parahaemolyticus*, which contains two T3SSs: T3SS1 and T3SS2. The *V. parahaemolyticus* T3SS2 is restricted to its pandemic serotype, O3:K6, and shares close sequence similarity to the T3SS first detected in *V. cholerae* [23]. T3SSs have been shown to be sufficient for intestinal colonization by non-7PET *V. cholerae* in infant mouse and rabbit models, even in the absence of TCP [23,25]. The presence and dynamic acquisition of these virulence factors demonstrate the disease-causing potential of non-7PET *V. cholerae*. However, due to the focus on surveillance for 7PET *V. cholerae*, little is known about the potential of non-7PET lineages as an emerging threat for future outbreaks [18,26].

In this study, 34 isolates from travellers returning from Indonesia to Australia between 2005 and 2017 provided an opportunity to look at the potential for long-range dissemination of different *V. cholerae* lineages. The isolates were whole genome sequenced, placed in phylogenetic context, and screened for genetic elements of interest such as antimicrobial resistance (AMR) genes and virulence factors. This study provides insight into the potential of a range of *V. cholerae* lineages for long-range carriage.

## Methods

### Biochemical testing and sequencing of bacterial isolates

*V. cholerae* isolates from patients returning from Indonesia to Australia presenting with symptomatic diarrhoea to primary pathology testing laboratories were referred to the Microbiological Diagnostic Unit Public Health Laboratory (MDU-PHL). Here, isolates were cultured for species confirmation, serotyping, biotyping and toxin gene detection.

The identity of these strains was first confirmed by growth on thiosulphate-citrate-bile salts-sucrose agar (TCBS, Oxoid) and a panel of biochemical tests [27]. The serogroup of these strains was subsequently confirmed using polyvalent O1, monovalent O1 (Inaba and Ogawa) and O139 agglutinating antisera (Mast Assure). All *V. cholera*e strains that serotyped as O1 were biotype tested for haemolysis of sheep erythrocytes and production of acetylmethylcarbinol (VP, Vogues–Proskauer), and submitted for PCR assays for the detection of the *V. cholerae* species-specific *ompW* and *ctxA* genes, as previously described [28,29]. These isolates underwent whole genome sequencing (WGS) on the Illumina NextSeq 500 platform with 150 bp paired-end reads (Supplementary Data, available in the online version of this article). Reads are available in BioProject PRJNA856407 from the NCBI Sequence Read Archive and individual accession IDs are listed in the Supplementary Data.

### Genome assembly and annotation

*De novo* sequence assembly was performed from paired-end short reads using Unicycler v0.5.0 [30] with default parameters. Contigs were then annotated with Prokka v1.14.5 [31]. Quality metrics for the generated assemblies and annotations were obtained using Quast v5.0.2 [32] and CheckM2 v1.0.1 [33], with thresholds of >99% completeness and <0.2% contamination. Pangenome analysis was performed with Panaroo v1.3.3 [34] from the annotated assemblies to generate a core gene alignment, with merged paralogues and a core gene threshold of 99%.

### Phylogenetic analysis

Published *V. cholerae* genomes obtained from the collection in Pathogenwatch (https://pathogen.watch/genomes/all?organismId=666, accessed 3 August 2023) were used as contextual sequences (Supplementary Data). Five representative isolates from each of the three global waves of 7PET transmission from its origin in the Bay of Bengal, selected based on time and geographical region of collection, and 868 non-7PET isolates across diverse clades were included. A pangenome was generated using Panaroo, as described above, with 883 contextual genomes and the 34 isolates from this study. The polymorphic sites (*n*=760 710 SNPs) from the resulting core gene alignment (*n*=2682 genes) were obtained using SNP-sites v2.5.1 [35] and used to build a maximum-likelihood (ML) phylogenetic tree using IQ-TREE v2.2.0 [36], with the GTR+F+ASC+R10 model (determined by first running model finder; -m MF) and 1000 ultrafast bootstraps.

An ML tree was also built for the 34 travel-associated isolates from this study based on polymorphic sites of the core gene alignment as described above. A mapped-SNP alignment to the N16961 7PET wave 1 reference (accession ID: GCA_000006745), made using SMALT v0.7.419 [37], was used to generate an ML tree for the 7PET isolates from this study and the five contextual 7PET isolates from each wave of transmission. All generated trees were visualized using iTOL v6.8.1 [38]. Pairwise SNP distances between isolates were calculated using SNP-dists v0.7.0 [39].

### Characterizing genes of interest

The presence of AMR genes was determined through a BLASTn-based method with Abricate v.1.0.1 [40] with default parameters using the Comprehensive Antibiotic Resistance Database (CARD, v.3.2.4) [41] on the whole genome assemblies of all 34 isolates in this study. ARIBA v2.14.6 [42] was then used with the same database on the read sets of all 34 isolates. Matches were designated in ARIBA as 95% of the reference gene sequence matching to the assembly and the gene assembling into one contig and matches in Abricate were those with 80% identity and 80% coverage compared to the reference gene sequence. The results from these two methods were combined to generate a profile of AMR gene presence.

The same analysis was conducted with both Abricate and ARIBA using the Virulence Factor Database (VFDB, accessed 13 October 2023) [43]. Abricate was also used with the plasmidfinder database to find putative plasmid sequences. Structural proteins and putative effectors were also identified for different bacterial secretion systems through this VFDB search, and the HMmer Based UndeRstandinG of gene clustERs (hamburger) tool was used to find clusters of structural genes for the T3SS [44]. A custom database of Hidden Markov Models (HMMs) was built with HMMer v.3.2.1 [45] from known T3SS structural genes (Supplementary Data) in representative isolates of *V. cholerae* (accession IDs: GCA_000153785.3, GCA_900538065.1, ERS2493980)*,* and close relatives *Vibrio parahaemolyticus* (accession IDs: GCA_000196095.1, AB455531.1) and *Vibrio anguillarum* (accession ID: GCA_002813795.1) for the three known types of T3SS in *V. cholerae*.

The *ctxB* genotype, which has been associated with different 7PET outbreaks, was identified by running BLASTn v.2.14.1 [46] on the whole genome assemblies for all 34 isolates with a custom database of representative sequences for each *ctxB* allele, using a 90% sequence identity and 70% gene coverage threshold. Presence of the SXT ICE was first explored through building a custom ARIBA database with representative sequences in *V. cholerae* (Supplementary Data) and running a search on the read sets of all 34 isolates as described above. A BLASTn search was then undertaken with the same database on the assemblies for matches that were found in partially assembled contigs by ARIBA.

## Results

### The majority of *V. cholerae* isolates from returning travellers to Australia come from non-7PET lineages

**Fig. 1.**
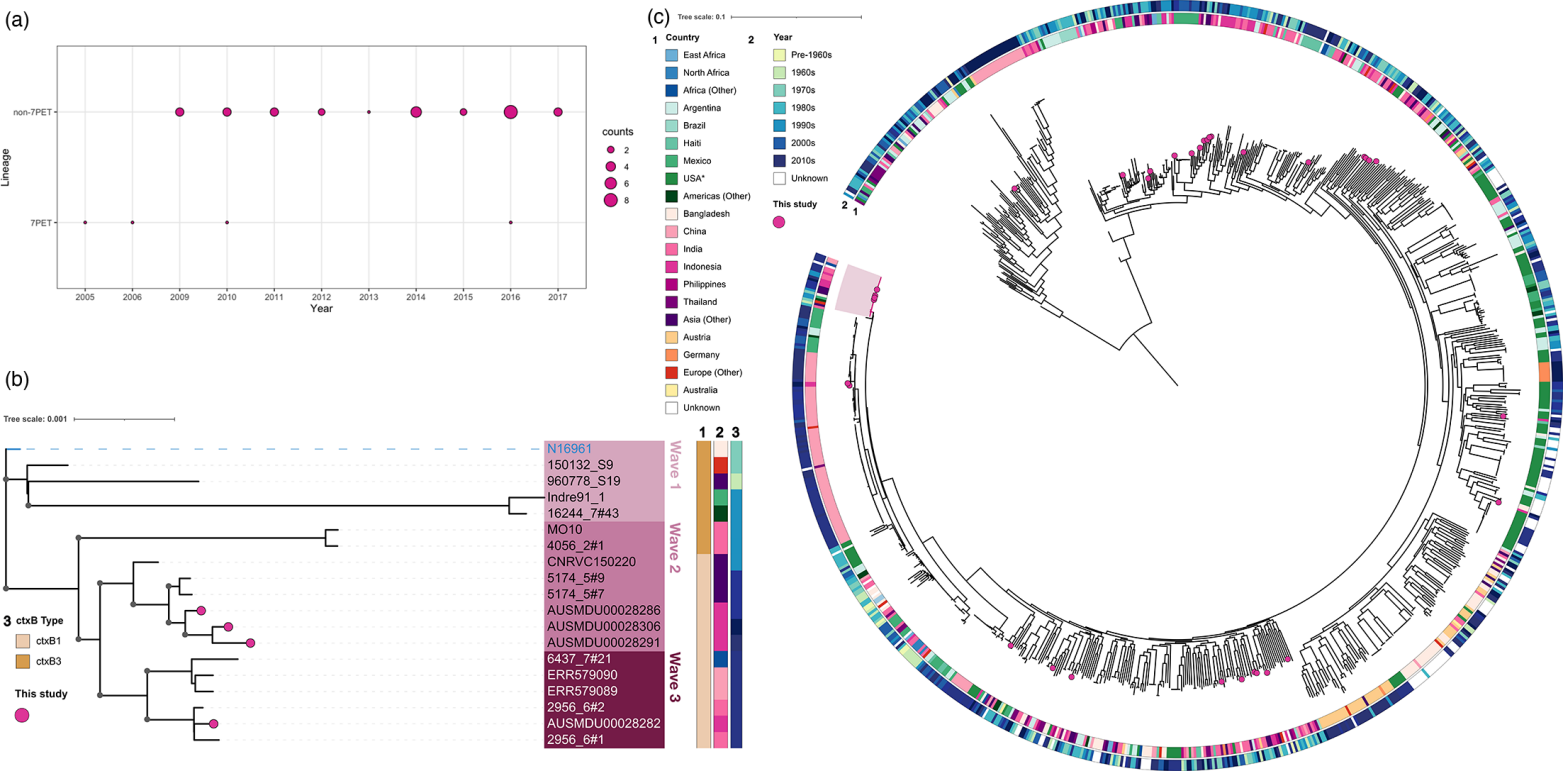
Isolates from returning travellers to Australia included both 7PET and non-7PET *V. cholerae*. (**a**) Temporal distribution of non-7PET and 7PET *V. cholerae* isolates in this study, with the size of the circles relative to the number of genomes collected for the given lineage or group of lineages in each year. (**b**) ML phylogenetic tree with 1000 ultrafast bootstraps for 19 7PET *V. cholerae* genomes, based on SNPs (*n*=2603 SNPs) of a core gene alignment (*n*=3187 genes), rooted on the *V. cholerae* strain N16961 (highlighted in blue). Bootstraps are indicated by grey circles for nodes with bootstrap values ≥90. Metadata for country, year of isolation and *in silico* identification of *ctxB* type are shown in coloured blocks (1, 2 and 3 respectively; see key). Isolates from each wave of transmission are denoted by the three different pink blocks. Isolates from this study are indicated by pink circles on the tree tips. (**c**) ML phylogenetic tree with 1000 ultrafast bootstraps for non-7PET genomes (*n*=868) and five representatives from each wave of 7PET transmission based on SNPs (*n*=760‍710 SNPs) of a core gene alignment (*n*=2682 genes), rooted on a clade that contains isolates which have been suggested to belong to *V. paracholerae*. Metadata for country (ring 1) and year of collection (ring 2) are shown for each isolate. The clade shaded in pink denotes the 7PET lineage. Isolates from this study are indicated by pink circles on the tree tips. Trees were visualized and annotated with iTOL v6.8.1.

The 34 *V*. *cholerae* isolates obtained between 2005 and 2017 from returning travellers who presented with diarrhoea in Australia (Fig. 1a) underwent WGS, revealing this collection comprised seven *V. cholerae* O1 Ogawa and 27 *V*. *cholerae* non-O1, non-O139. *In silico* analysis for presence of the 7PET lineage-specific marker gene *VC2346* showed that four isolates carried this gene, indicating they belonged to the 7PET lineage (Fig. 1b), while the remaining 30 isolates belonged to non-7PET lineages.

### Phylogenetic analysis reveals diversity of *V. cholerae* in Indonesia

Given that most isolates were from non-7PET lineages, we inferred the phylogeny of the isolates sequenced here in the context of a large collection of published non-7PET genomes (*n*=868), as well as five representatives from each wave of 7PET transmission, based on SNPs (*n*=760 710 SNPs) of a core gene alignment (*n*=2682 genes). The contextual sequences included isolates from between 1916 and 2020 and across 49 countries in six continents, representing the known diversity of the species (Fig. 1c). Four of the isolates from this study were from the 7PET lineage and, of these, three (AUSMDU00028286, AUSMDU00028291, AUSMDU0028306) fell within wave 2 and one (AUSMDU00028282) within wave 3 of 7PET transmission (Fig. 1b). All four carried the *ctxB1* genotype of *ctxB*. The wave 2 isolates clustered with the Global 2B sublineage, and the wave 3 isolate with the Global 3B sublineage [26,47]. Both sublineages have previously been described as having geographical signatures of predominantly Asian origin and dominant *ctxB1* genotypes (Fig. 1b) [26].

**Fig. 2.**
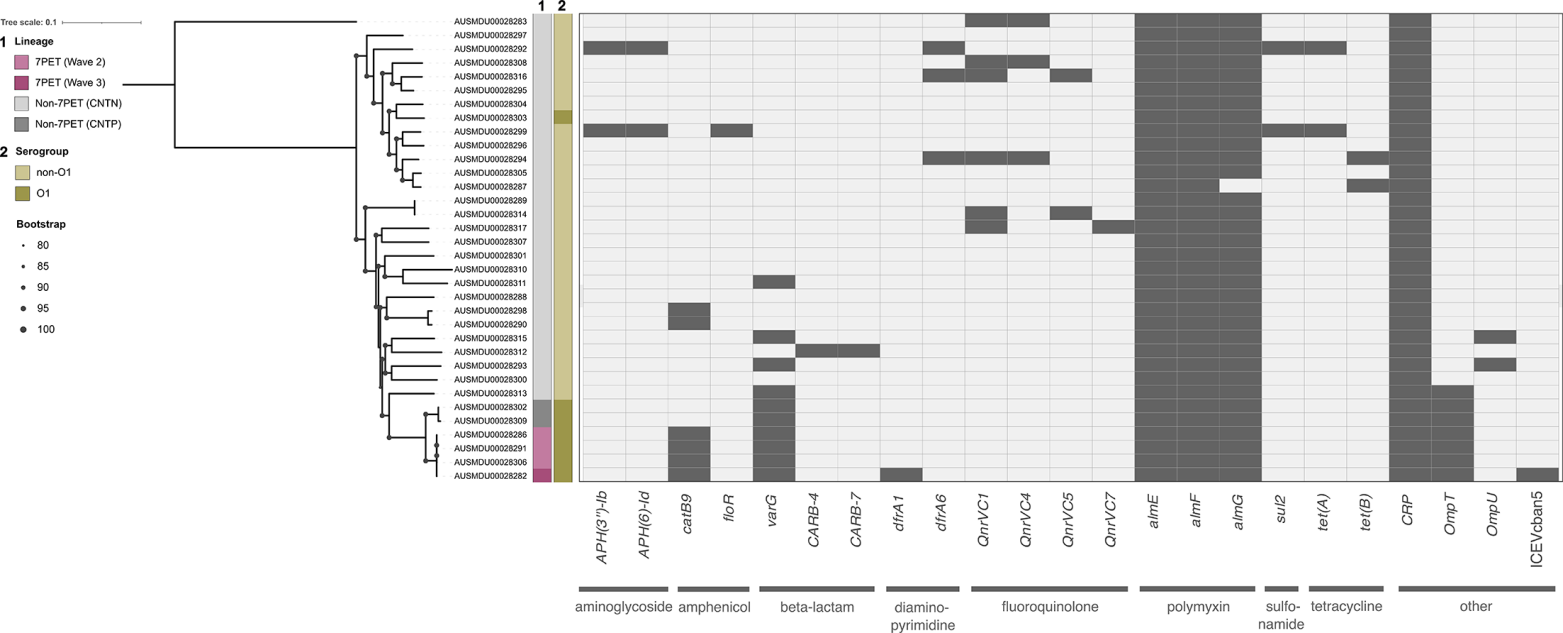
AMR genes present in travel-associated *V. cholerae* isolates. ML phylogenetic tree with 1000 ultrafast bootstraps for the 34 *V*. *cholerae* genomes from this study, based on SNPs (*n*=271 209 SNPs) of a core gene alignment (*n*=2833 genes), rooted on the AUSMDU00028283 isolate from this study. Metadata for the lineage and *in silico* identification of serogroup are shown. Presence (dark grey) and absence (light grey) of AMR genes for each isolate are shown, detected through a blast-based search of the CARD, with the antibiotic class that each gene confers resistance to indicated with grey bars underneath.

The 30 non-7PET isolates from this study fell across diverse phylogenetic branches (Fig. 1c). The mean number of SNPs among these non-7PET isolates was 52 845 SNPs (minimum: 55, maximum: 120 204), compared to 73 SNPs (minimum: 20, maximum: 129) among the 7PET isolates from this study, consistent with the knowledge that the 7PET lineage is clonal. None of these non-7PET genomes clustered with each other, except for two CNTP isolates (Fig. 2). These CNTP isolates differed from each other by 1585 SNPs. The mean SNP distance among these two isolates and the 28 other non-7PET isolates from this study was 54 599 SNPs. In contrast, the mean SNP distance among these CNTP isolates and the four 7PET isolates from this study was 15 343 SNPs, suggesting that they were more genetically similar to 7PET *V. cholerae*. The rest of the isolates did not cluster together, nor with any of the non-7PET clusters that have been previously linked to outbreaks. This suggests that *V. cholerae* isolated from returning travellers in this study were phylogenetically distinct both from each other and from previously observed non-7PET *V. cholerae*.

### Travel-related *V. cholerae* isolates carried various AMR genes

Given that many of the travel-related isolates sequenced here were phylogenetically distinct, next we wanted to determine if they were also genotypically distinct. To do this, we first considered their AMR profiles. We looked for the SXT ICE to assess if presence of this element and its associated AMR genes were concordant with the waves of transmission in which our 7PET isolates fell. However, none of the wave 2 isolates in this study contained a complete SXT ICE. Only the wave 3 isolate (AUSMDU00028282) carried a sequence similar to the *V. cholerae* Ban5 ICE, ICEVchban5 (accession number GQ463140.1). This isolate was also the only one that encoded *dfrA1*, which is carried on ICEVchban5. One of the non-7PET isolates, AUSMDU00028299, carried *floR*, *sul2* and *tetA* genes, known to confer resistance to amphenicol, sulfonamide and tetracycline antibiotics, respectively. These were encoded on the same contig in a region that contained a partial match to the *V. cholerae* IDH_1986 strain SXT ICE. We also found one non-7PET isolate (AUSMDU00028290) with a partial match to the *Aeromonas hydrophila* IncU plasmid pRA3 (accession ID: DQ401103), spanning 565 bp with 99.8% nucleotide sequence identity.

We then looked for all other known AMR genes and found that these were sporadically present across both 7PET and non-7PET lineages. The *catB9* gene was detected in all four 7PET as well as two non-7PET isolates (Fig. 2). In contrast, some AMR genes were more conserved across the dataset. All but one of the isolates from this study carried the *almEFG* operon, while the other isolate carried *almE* and *almF* but not *almG*, which is the glycyltransferase primarily responsible for antimicrobial activity [48]. AlmEFG act together to modify lipid A with glycine or diglycine, conferring polymyxin B resistance. Polymyxin antibiotics are often used as a last line strategy against MDR Gram-negative bacteria [49]. Interestingly, the 7PET lineage, but not classical biotype O1, has previously been found to be polymyxin resistant [50]. Other AMR genes were sporadically present across the non-7PET isolates including *tetA* and *tetB*, which encode resistance to tetracycline antibiotics, and *qnrVC1*, *qnrVC4*, *qnrVC5* and *qnrVC7* genes for quinolone resistance (Fig. 2). These are of particular interest as tetracycline and fluoroquinolone antibiotics are recommended by the WHO for the treatment of cholera [51].

### Virulence factors were detected in both 7PET and non-7PET isolates

**Fig. 3.**
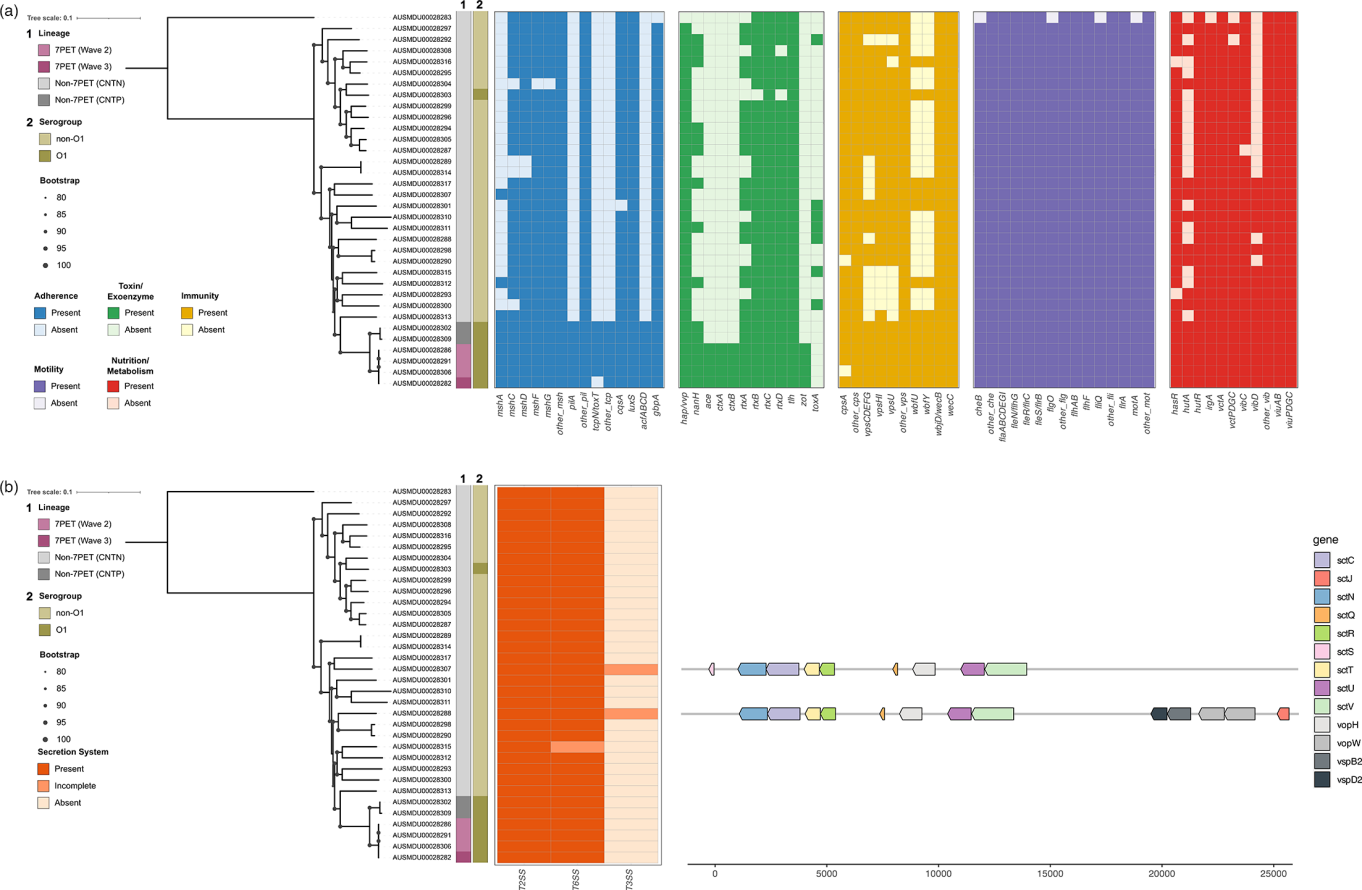
Genes associated with virulence present in travel-associated *V. cholerae* isolates. (**a**) ML phylogenetic tree with 1000 ultrafast bootstraps for the 34 *V*. *cholerae* genomes from this study, based on SNPs (*n*=271 209 SNPs) of a core gene alignment (*n*=2833 genes), rooted on AUSMDU00028283 from this study. Metadata for the lineage and *in silico* identification of serogroup are shown. Presence (dark) and absence (light) of virulence factor genes for each isolate are shown, detected through a blast-based search of the VFDB, where each block is coloured by functional category. Genes in an operon that are present in all or the same subset of isolates are denoted as ‘other’ (full list of genes in Supplementary Data). (**b**) The same ML phylogeny, with presence, absence or incomplete presence of secretion systems shown, where incomplete systems are those that lack at least one structural component. The operons of structural genes, detected through HMM-based methods for the two T3SS-positive isolates from this study, are shown.

Next, we screened for genetic determinants linked to virulence across different functional classes. The only genes that were present exclusively in 7PET isolates were those for the toxin genes *ctxAB,* accessory enterotoxin (*ace*) and zonula occludens toxin (*zot*) (Fig. 3a). These genes encode the primary toxins involved in 7PET *V. cholerae* pathogenicity and are all present together in a region in the core of the CTXφ phage called the virulence cassette. Zot modifies tight junctions to increase permeability of intestinal mucosa, while Ace potentially acts as an ion-transporting ATPase but its function is less well understood [52].

Genes for TCP biosynthesis and assembly, which are responsible for the primary mechanism of intestinal colonization by 7PET *V. cholerae*, were found in all four 7PET isolates as well as the two CNTP non-7PET isolates. These genes are clustered together in several transcriptional units: *tcpI, tcpP-H, tcpA-F* and *toxT-tcpJ* [53]. All six isolates contained the complete set of TCP genes, with the exception of *tcpN*/*toxT* that was found in five of these six isolates. The *tcpA* gene encodes the major structural subunit of TCP, and *tcpB* encodes the minor pilin gene [54]. These isolates also had other *tcp* genes such as: the putative outer membrane (OM) porin TcpF, which may form an OM channel where TcpA is extruded; the ATP-binding protein TcpT; TcpE that anchors TcpT to the cytoplasmic membrane; and the TcpJ pilin signal peptidase that processes TcpA [53]. *TcpN*/*toxT* is a regulatory gene that controls the transcription of TCP and other key virulence factors such as CTX.

From a blast-based search of the VFDB [43], most of the virulence factors detected were present across all of the isolates in this study. Genes for adherence (*msh*, *pil*), toxins and exoenzymes (*rtx*, *hap*/*vvp*)*,* immunity (*cps, vps*, *wbf*) and for protective metabolism (*vctA/PDGC, vib*) were present in nearly all of the isolates. Operons encoding motility genes (*che, fla, fle, flg, flh, fli, mot*) were also conserved, and components of these clusters of motility genes, namely *cheB, flgO, fliQ* and *motA,* were absent from only one non-7PET isolate (Fig. 3a).

Structural components and effectors of type 2 and type 6 secretion systems (T2SS, T6SS) were found in all 34 isolates through the VFDB search. The T2SS has been previously observed to be constitutively expressed in *V. cholerae* at 37 °C, with a role in both virulence and environmental survival [55]. The T6SS has been found in all sequenced *V. cholerae* isolates and is responsible for the translocation of effectors into both eukaryotic and prokaryotic cells in a contact-dependent manner [56]. T3SS components were not found through the blast-based VFDB search. Hence, as these are difficult to detect through sequence identity-based methods due to close similarity of many structural components to flagellar proteins [57], we performed an HMM-based search for T3SS clusters. This revealed that only two non-7PET isolates (AUSMDU00028288, AUSMDU00028307) contained structural components for the T3SS, consistent with previous studies that only found the T3SS is non-7PET *V. cholerae* [58]. Both isolates were non-toxigenic and TCP-negative, and both T3SSs found included secretin, which unlike the other components, does not have homologues in flagellar proteins [2159^21^]. Both structures also had conserved presence and order of structural gene components but were each predicted to be incomplete compared to the included references (Fig. 3b).

## Discussion

Here we report the nature and diversity of genomes generated from 34 *V*. *cholerae* isolates, taken from travellers returning to Australia from Indonesia. Four of these belong to the 7PET lineage. As there is little publicly available data on cholera incidence in Indonesia, and, of the years covered by our samples, the WHO only reported statistics in 2005 (1 338 cases), it is difficult to determine if these 7PET isolates coincided with peaks in domestic cases. However, one isolate was obtained in 2010, when a cholera outbreak occurred in four Indonesian provinces and totalled 2160 cases [59]. The other 30 isolates were from diverse non-7PET lineages. Only the two CNTP non-7PET isolates clustered together. These were collected in 2014 and 2016, suggesting this lineage was present for at least these two years, and placed within a cluster that was associated with the previously described L3b lineage that also comprised CNTP isolates and caused an outbreak in Hangzhou, China, between 2001 and 2012 [18]. The other non-7PET isolates did not cluster with any previously observed lineages.

The observed trends in presence of AMR genes corresponded both with previous knowledge of AMR in *V. cholerae* and with antibiotic treatment recommendations by the WHO [51,60]. Some of the AMR genes found are also known to be encoded on genomic islands. One of the non-7PET isolates in this study contained a partial match to the pRA3 plasmid, although only to a 565 bp region that did not map to any of the AMR genes (*sul1*, *aadA2*, *catA2*) previously described in pRA3 [61]. The *catB9* gene, which was found only in the four 7PET and two of the non-7PET isolates from this study, encodes a chloramphenicol acetyltransferase gene conferring low-level phenicol resistance. The *catB* cassette is encoded within the superintegron (SI) in chromosome 2 of *V. cholerae*, which itself allows for the capture and excision of different gene cassettes through site-specific recombination. The *catB9* gene is often silent in 7PET isolates due to its distance from a promoter [62,63]. However, previous experimental studies have shown that integrase-mediated rearrangement of gene cassettes within the SI region can result in the relocation of *catB* closer to the promoter, leading to induction of chloramphenicol resistance [63]. Similarly, two members of the CARB family of carbenicillin-inactivating β-lactamases, CARB-4 and CARB-7, were found in one of the non-7PET isolates in this study. The *bla*_CARB-7_ gene cassette has previously been found in non-7PET *V. cholerae*, flanked by *V. cholerae* repeat regions contained within the SI, although was not able to be transferred through conjugation [64]. In contrast, the *bla*_CARB-4_ gene cassette is associated with the pUD12 plasmid in *Pseudomonas aeruginosa* and includes integrons in its flanking sequences [65,66]. The *tetA* gene, which was found in two non-7PET isolates from this study, has also been found on MGEs such as in an IncA/C2 plasmid in *V. cholerae* [67] or in the *Salmonella* genomic island 1 [68]. Altogether, the results demonstrate the presence of diverse AMR genes in our sequenced isolates, with some sporadically distributed across non-7PET lineages and none of these genes unique to the 7PET lineage.

The presence of genes associated with virulence also highlighted the genetic diversity of these isolates. TCP was detected in the 7PET isolates and in two CNTP non-7PET isolates, but is typically associated with 7PET *V. cholerae*, where it drives intestinal colonization and thus pathogenesis. Genes for TCP biosynthesis have previously been found in non-7PET outbreak clades, even in isolates without CTXφ, such as the ELA-3, Gulf Coast and MX-2 lineages that have been associated with disease in the Americas between the 1970s and the 2010s [6]. Like the two CNTP isolates from this study, these lineages belong to the O1 serogroup, which is predominantly associated with pandemic cholera. The L3b (2001–2012) and L9 (2013–2018) non-7PET lineages responsible for cholera outbreaks in Hangzhou, China, were primarily CNTP, with CTXφ acquisition observed in 21% of collected isolates [18]. Moreover, the two CNTP isolates and the four 7PET isolates in this study had accessory colonization factor genes *acfABCD*, which like TCP are encoded on *V. cholerae* pathogenicity island 1, and have been shown in O1 classical biotype isolates to have a role in colonization of the mouse intestine [69].

Canonical *V. cholerae* toxin genes *ctxAB, zot* and *ace* were only detected in the 7PET isolates in this study, but other toxin and exoenzyme genes such as *rtx* and *nanH* were present in both 7PET and non-7PET isolates. The *rtxABCD* operon confers cytotoxic activity, with *rtxA* encoding a toxin that resembles repeats-in-toxin family exotoxins, while the *nanH* neuraminidase is able to unmask additional GM1 receptors for CTX, increasing binding and penetration and therefore severity of disease [70,72]. The T3SS is thought to be sufficient for both intestinal colonization and inflammatory diarrhoea even in the absence of TCP and CTX [23]. Here, the T3SS was not detected in 7PET isolates, consistent with previous literature [58], and in only two of 30 non-7PET isolates in this study, suggesting that this was not the primary virulence determinant of non-7PET * V. cholerae* found in this study.

Future research is required to assess the distribution and role of these virulence factors in establishing disease. This would require additional non-7PET *V. cholerae* genomes in order to undertake larger-scale comparative genomic analysis. For example, genome-wide association studies could further elucidate relationships between the presence of known or novel virulence-associated genes across lineages and disease presentation or risk. Methods to accurately determine the serogroup *in silico* would also be essential for understanding the prevalence of different disease-causing non-O1, non-O139 lineages. Although the genetic context of the AMR genes found was not the focus of this study, one limitation of short-read sequencing data is that it is not possible to assemble complete plasmid or integron sequences. Notwithstanding this, we only found one plasmid linked to AMR genes by running the plasmidfinder database. Further work would be required to understand the detailed genomic context associated with such elements carrying AMR genes.

Nonetheless, through phylogenomic approaches, this study illustrates the potential for long-range carriage of a diverse selection of both 7PET and non-7PET *V. cholerae*. The carriage of *V. cholerae* through international travel has been previously described, for example to Australia (1972–2011) [73], Russia (2005–2012) [74] and the UK (2004–2018) [75]. In the UK, those with recent travel to cholera-endemic countries and who present with diarrhoea are recommended to test for * V. cholerae* according to the Investigation of Faecal Specimens for Enteric Pathogens, as per the UK Standards for Microbiology Investigations. Biochemical analysis of 836 *V*. *cholerae* isolates from the UK Health Security Agency collection revealed that 792 of these were associated with recent travel history abroad, and similar to this study, most of the isolates were non-O1, non-O139 (623/836, 74.5%) [75]. While we note that there are potential sampling biases in the referred Australian isolates, and travel was limited to country of reported travel, these data still enable the detailed exploration of the phylogenetic and genomic diversity of such travel-associated isolates. The distribution of the non-7PET isolates from this study across diverse branches of the global phylogeny demonstrates the highly diverse nature of these isolates, both from each other and from previously described non-7PET outbreak lineages. Most isolates collected in this study were from non-7PET lineages that carried multiple virulence factors and resistance genes against antibiotics that are commonly used against cholera, reinforcing the knowledge that non-7PET lineages are able to cause disease, although through different mechanisms than the 7PET lineage. Thus, WGS of travel-associated isolates from globally representative sources is important for generating a more complete picture of cholera diversity, pathogenicity and transmission dynamics.

## supplementary material

10.1099/mgen.0.001307 Uncited Supplementary Data Sheet 1.

## Abbreviations

AMR: antimicrobial resistance
CARD: comprehensive antibiotic resistance database
CNTP: CTX-negative, TCP-positive
CTX: cholera toxin
HMM: Hidden Markov Model
ICE: integrative conjugative element
MDR: multi-drug resistance
MGE: mobile genetic element
ML: maximum-likelihood
OM: outer membrane
7PET: seventh pandemic El Tor
SI: superintegron
SXT: sulfamethoxazole–trimethoprim
TCP: toxin coregulated pilus
T2SS: type 2 secretion system
T3SS: type 3 secretion system
T6SS: type 6 secretion system
VFDB: virulence factor database
WGS: whole genome sequencing
WHO: World Health Organization
